# The Extent of Gender Gap in Citations in Ophthalmology Literature

**DOI:** 10.3389/fmed.2022.855385

**Published:** 2022-05-18

**Authors:** Suqi Cao, Yue Xiong, Wenhua Zhang, Jiawei Zhou, Zhifen He

**Affiliations:** Department of Ophthalmology, Eye Hospital, Wenzhou Medical University, Wenzhou, China

**Keywords:** gender, equity, ophthalmology, citation, generalized additive model

## Abstract

**Purpose:**

To investigate the severity and causes of gender imbalance in the counts of ophthalmology citations.

**Methods:**

The PubMed database was searched to identify cited papers that were published in four journals (*Prog Retin Eye Res*, *Ophthalmology*, *JAMA Ophthalmol*, and *Invest Ophthalmol Vis Sci*) between August 2015 and July 2020, and those that referenced these cited papers by 2021 July (i.e., citing papers). The gender category of a given paper is defined by the gender of the first and last author (MM, FM, MF, and FF; M means male and F means female). A generalized additive model to predict the expected proportion was fitted. The difference between the observed proportion and expected proportion of citations of a paper’s gender category was the primary outcome.

**Results:**

The proportion of female-led (MF and FF) papers slightly increased from 27% in 2015 to 30% in 2020. MM, FM, MF, and FF papers were cited as −9.3, −1.5, 13.0, and 23.9% more than expected, respectively. MM papers cited 13.9% more male-led (MM and FM) papers than female-led papers, and FF papers cited 33.5% fewer male-led papers than female-led papers. The difference between the observed proportion and expected proportion of MM citing papers within male-led and female-led cited papers grew at a rate of 0.13 and 0.67% per year.

**Conclusion:**

The high frequency of citations of female-led papers might narrow the gender gap in the citation count within ophthalmology. These findings show that papers by female-led are less common, so the gender gap might still exist even with their high citation count.

## Introduction

Women have faced societal pressures and barriers associated with gender (1–3) compared to men. For this reason, scientists have become concerned with the gender imbalance in academia (4), such as women have won fewer awards (5), published fewer papers (6), and accumulated fewer citation counts (7) even if they comprise of more than 50% Ph.D. holders in America. Accumulating evidence shows that women have been underrepresented (7), especially in the fields of science, technology, engineering, mathematics, and medicine (STEMM) (8).

Despite an increase in the proportion of female graduates in medical school (9) and the proportion of female authors in medicine (10), the representation of female ophthalmologists in academic medicine has been much lower than male ophthalmologists (11). Gender imbalance has manifested in the male/female proportion of authors in papers within ophthalmology. To illustrate, Heng Wong et al. (12) analyzed the top 100 cited papers in ophthalmology from 1975 to 2017 and found that 70% of the first authors were male. In addition, the gender imbalance can be observed not just in the proportion of authors in scientific papers but also in academic ranks, such as senior professorships (13), leadership positions (4, 9, 11), and participation in reputable conferences (14). Some have spearheaded efforts to mitigate the gender imbalance against women. For instance, Dr. Mariya Moosajee, Dr. Julie Daniels, and Dr. Maryse Bailly established the Women in Vision UK (WVUK) network to mitigate gender inequality (15).

Assessment criteria for performance in academia include academic ranks, peer-reviewed publications, salary, and funding (3, 16, 17). The number of peer-reviewed publications has been especially important in climbing up the ladder of academic ranks (9, 18–20) and securing funding for principal investigators. A recent study by Dworkin et al. (21) examines the severity of gender imbalance in the citation count of neuroscience papers. They analyzed 303,886 articles that were published in five top neuroscience journals between 1995 and 2018 and examined the link between authors’ gender and citations. They revealed that female authors have received fewer citations than expected and that this gender imbalance might not be alleviated over time.

As mentioned above, several previous studies on gender bias in ophthalmology focus on the percentage of female authors, female academic ranks, and citation count, all of which mainly measure the passive consequences of gender behavior. Using the framework of the relationship between authors’ gender and the gender makeup of their citation lists (21), one could directly measure the citation behavior itself. In our study, we were interested in investigating the severity of gender imbalance in the citation count of ophthalmology papers in ophthalmology citations. To do so, we analyzed papers published between August 2015 and July 2020 in four top ophthalmology journals (*Prog Retin Eye Res, Ophthalmology, JAMA Ophthalmol*, and *Invest Ophthalmol Vis Sci*), which had the highest h-index (22) in 2020.

## Materials and Methods

### Data Collection

**FIGURE 1:**
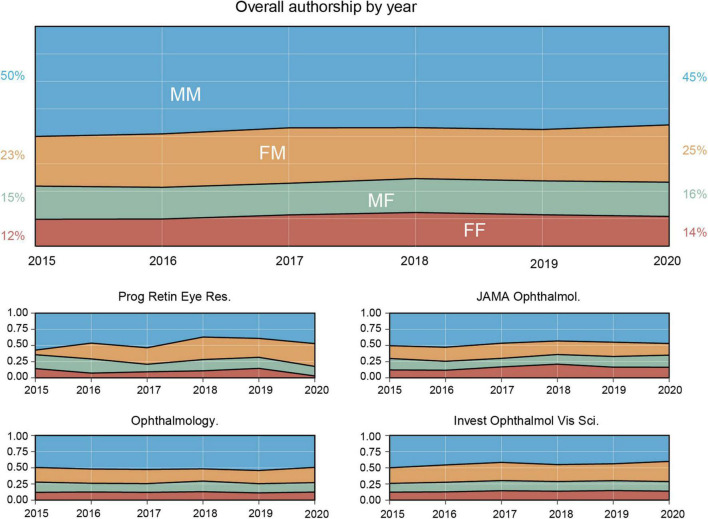
Trends of gender among authors within the four ophthalmology journals between August 2015 and July 2020. Proportions of published papers with male as first and last authors (MM, blue), female as the first author and male as the last author (FM, yellow), male as the first author and female as the last author (MF, green), and female as the first and last authors (FF, red) are plotted as a function of time (between August 2015 and July 2020) in four ophthalmology journals: *Prog Retin Eye Res*, *Ophthalmology*, *JAMA Ophthalmol*, and *Invest Ophthalmol Vis Sci*. The overall trend of these four journals is also plotted (top panel).

**FIGURE 2:**
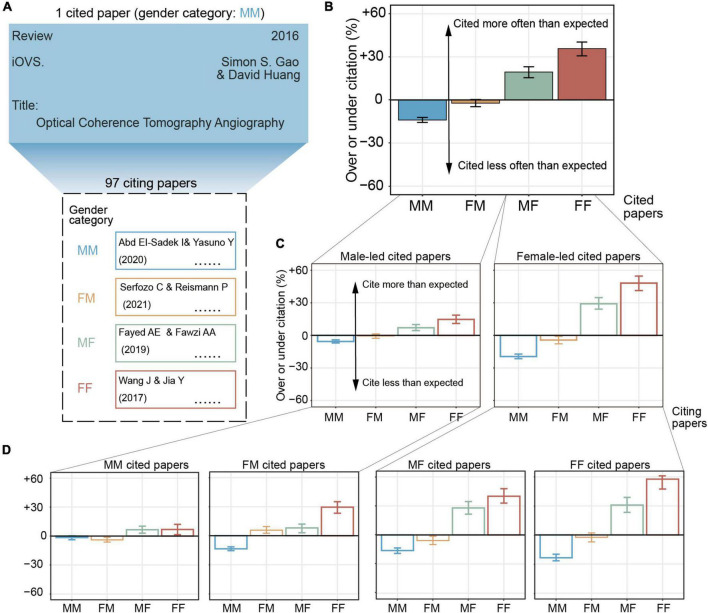
The gender gap in citation counts. **(A)** Definition of cited paper and citing papers. Gender category of a given paper is defined by the gender of the first and last author. In the example we provide here, a review paper from Dr. Simon was published in iOVS in 2016. We defined it as an MM cited paper using the GenderAPI database. This cited paper has been cited by 97 citing papers. **(B)** The degree of over- or under-citation of different gender category of cited paper. MM papers were cited 9.3% less than expected (95% CI, –10.6 to –8.0%), FM papers were cited 1.5% less than expected (95% CI, –3.1 to 0.5%), MF papers were cited 13.0% more than expected (95% CI, 10.5–15.2%), and FF papers were cited 23.9% more than expected (95% CI, 20.5–27.2%). **(C)** The degree of over- or under-citation after separating cited papers by the more common (i.e., led) gender and **(D)** by full gender. Bars represent overall over-citation and under-citation, calculated from 53,962 total citation counts (MM, 24,058, FM, 13,742, MF, 8,384, and FF, 7,778). Error bars represent the 95% of CI of each citation estimate, calculated from 1,000 bootstrap resampling iterations.

We have selected three research journals (*Ophthalmology*: 244 [h-index]; *Invest Ophthalmol Vis Sci*: 218; and *JAMA Ophthalmol*: 196) and one review journal (*Prog Retin Eye Res*: 152) with the highest h-index in the ophthalmology field in 2020 (Figure 1). We searched the PubMed database for papers published from August 2015 to July 2020 in these four journals and defined these papers as cited papers. Papers that referenced these cited papers by July 2021 were defined as citing papers (Figure 2A). We obtained the Author Full Name (AF), Source of Publication (SO), Document Type (DT), Publication Date (PD), and Published Year (PY) for each cited paper. We also searched PMID of Cited by lists (CL) and Times Cited Count (TC) of above-cited papers and obtained the AF, PD, and PY for each citing paper (the paper in CL) by July 2021.

### Gender Determination

Similar to Dworkin et al. (21), gender was awarded using a publicly available probabilistic database (GenderAPI).^1^ We attributed male (or female) to each author whose name had at least 85% probability of belonging to someone labeled as male (or female) according to the GenderAPI. We randomly selected 100 unique authors involved in the aforementioned dataset that we collected from PubMed for the manual gender verification and found that the accuracy of GenderAPI program was 98% (see Supplementary Material). In the current study, the gender of both the first and last author of 86% of the papers (both cited and citing papers) could be determined by using GenderAPI. Subsequently, we manually determined the gender of the authors by visiting lab websites for the remaining 14% papers.

### Self-Citations Removal

We defined self-citation papers where either the first or last author of the citing paper was the first or last author of the cited paper. In this study, self-citations were eliminated from all analyses of gender citation behavior.

### Statistical Analysis

To obtain an expected proportion that accounts for various characteristics that might be associated with gender, we fitted the same generalized additive model (GAM) as Dworkin et al. (21) on the multinomial outcome [MM (first and last authors are male), FM (first author is female and last author is male), MF (first author is male and last author is female), and FF (first and last authors are female)]. This model includes the following explanatory variables: (1) date (PD and PY), (2) team size (The number of authors), (3) source of publication (SO), (4) team seniority estimated with (TC), and (5) document type (DT). Then, we applied the model to each paper using the *mgcv* package in R (23), which returned the expected proportions of citing papers (MM, FM, MF, and FF) for a given cited paper. We then compared this expected proportion with the observed proportion of citations of the paper. If the expected proportion does not match, it means that the gender gap still exists after the consideration of the abovementioned variables of each paper.

In this study, we presented all estimations with a CI (95% confidence interval), a *p-*value, or both. The CIs were computed by bootstrapping the cited papers (e.g., randomly sampling 500 cited papers each time to get the average expected proportion for each iteration). Randomization was conducted by probabilistically drawing new gender categories for each paper according to their estimated gender probabilities by GAM. The statistical significance was adjusted for multiple comparisons; *p-*values were corrected according to the Holm–Bonferroni method (24).

Generally, the last author of a paper was considered the senior investigator. We defined a female-led (MF and FF) paper as the article in which the last author is female and a male-led (MM and FM) paper as the article in which the last author is male.

### Hypotheses

In this study, we tested four hypotheses:

*Hypothesis 1: The citation rate of female-led papers is lower than expected.*

To verify this assumption, we first estimated the expected proportion of citations given to each category of authors. This expectation was calculated by summing the probabilities estimated by the GAM for all papers from 2015 to 2020. These values were compared by calculating the percentage difference between observed and expected proportions for each author’s gender group. If the hypothesis is true, the percentage difference of female-led papers will be less than 0.

*Hypothesis 2: The citation of female-led papers occurs to a fewer degree in MM papers.*

We used a similar method to those described above to test the second hypothesis. The primary difference is that, instead of calculating the observed and expected proportion by summing over the citations within all citing papers, we performed those summations separately for lists in papers with male-led papers and female-led papers. If this hypothesis is true, MM papers will be citing more male-led papers than female-led papers.

*Hypothesis 3: The proportion of MM citations of female-led papers will be decreasing more than that of male-led papers over time.*

The changes in male-led papers and female-led papers over time were estimated using linear regression. The CIs of this estimate was obtained using the article bootstrap procedure, and significance was assessed using the graph-preserving null model (21). If the hypothesis is true, the annual growth rate of MM citation count from female-led papers will be lower than that of male-led papers.

*Hypothesis 4: A relationship exists between local co-authorship networks and citation behavior.*

A co-authorship network of first and last authors is defined as where they established a connection by co-authoring a paper with another author before a given date. We examined how the citation behavior was affected by the co-authorship network. If the hypothesis is true, there will be a consistent citation behavior between with co-authorship networks and without networks.

## Results

### Data Description

Our data included 8,084 cited papers, which were published in *Prog Retin Eye Res* (219 papers), *Ophthalmology* (2,265 papers), *JAMA Ophthalmol* (1,918 papers), and *Invest Ophthalmol Vis Sci* (3,682 papers) from August 2015 to July 2020 (data from the PubMed database). From those, 3,813 were MM papers (47.17%), 1,950 were FM papers (24.12%), 1,209 were MF papers (14.95%), and 1,112 were FF papers (13.76%).

### Authorship’s Trends

The proportion of female-led papers slightly increased from 27% in 2015 to 30% in 2020. On one hand, this trend of female-led papers varied across journals. To illustrate, *Prog Retin Eye Res* decreased from 35 to 18%*; Ophthalmology* hardly changed (from 28 to 27%)*; JAMA Ophthalmol* increased from 30 to 34%, and *Invest Ophthalmol Vis Sci* increased from 26 to 29%. On the other hand, the overall proportion of articles that had females as first or last authors slightly increased from 50% in 2015 to 55% in 2020 (Figure 1).

### Citation Imbalance

To test the extent of the gender gap in the number of citations, we narrowed the 8,084 cited papers to 5,864, which were either research or review articles (with at least 1 citation count) that were published in the four ophthalmology journals. We found that these 5,864 papers had been cited by 53,962 times before August 2021. We then obtained the observed proportion in each gender category for all 5,864 papers [e.g., a given cited paper was cited by 97 (47 MM, 27 FM, 22 MF, and 1 FF) citing papers, the observed proportion was 0.48, 0.28, 0.23, and 0.01, respectively; Figure 2A].

We studied whether there were any relationships between gender and paper characteristics (e.g., date, journal, team size, author seniority, and document type). We modeled the multinomial gender category (MM, FM, MF, and FF) as a function of the above characteristics by fitting a GAM, by which we estimated the expected proportion of citing papers for a given cited paper (Figure 2B). For all 5,864 cited papers, MM papers received 42.4% citations of observed proportion, compared to 25.9% for FM papers, 17.3% for MF papers, and 14.4% for FF papers. According to the relevant proportion of paper, the expected proportions were 46.8% (MM), 26.3% (FM), 15.3% (MF), and 11.6% (FF). Therefore, MM papers were cited 9.3% less than expected (95% CI, −10.6 to −8.0%), FM papers were cited 1.5% less than expected (95% CI, −3.1 to 0.5%), MF papers were cited 13.0% more than expected (95% CI, 10.5–15.2%), and FF papers were cited 23.9% more than expected (95% CI, 20.5–27.2%). The over-citation of MF papers and FF papers does not support the hypothesis that the citation rate of female-led papers is lower than expected (*Hypothesis 1*).

### The Effect of Author Gender on Citation Behavior

Among the abovementioned 5,864 cited papers that we screened, there were roughly 71% male-led papers and 29% female-led papers. In this section, after separating cited papers by gender, we found that, within male-led cited papers (TC: 37800), MM citing papers were cited 5.6% less than expected (95% CI, −7.0 to −4.1%, *p* < 0.001), FM papers were cited 0.5% less than expected (95% CI, −2.7 to 1.3%, *p* = 0.61), MF papers were cited 7.1% more than expected (95% CI, 4.5–10.0%, *p* < 0.001), and FF papers were cited 14.7% more than expected (95% CI, 11.0–18.7%, *p* < 0.001); within female-led cited papers (TC: 16162), MM papers were cited 19.5% less than expected (95% CI, −21.5 to −17.3%, *p* < 0.001), FM papers were cited 4.3% less than expected (95% CI, −7.8 to −1.0%, *p* = 0.01), MF papers were cited 29.1% more than expected (95% CI, 24.2–34.8%, *p* < 0.001), and FF papers were cited 48.2% more than expected (95% CI, 41.3–54.6%, *p* < 0.001; Figure 2C). Our results indicate that MM papers tended to cite fewer female-led papers (*Hypothesis 2*), whereas FF papers tended to cite more female-led papers.

Within the male-led group and female-led group, the citation proportion of MM and FM and MF and FF subgroups are plotted in Figure 2D. Specifically, the over-citation degrees of MM, FM, MF, and FF citing papers that cite MM cited papers gradually decreased, whereas those of MF and FF cited papers gradually increased.

### Time-Trends of Citation Imbalance

In addition to the overall citation behavior, we also quantified temporal trends of citation imbalance. We examined the yearly gap between the observed and expected proportions of MM citing papers.

**FIGURE 3:**
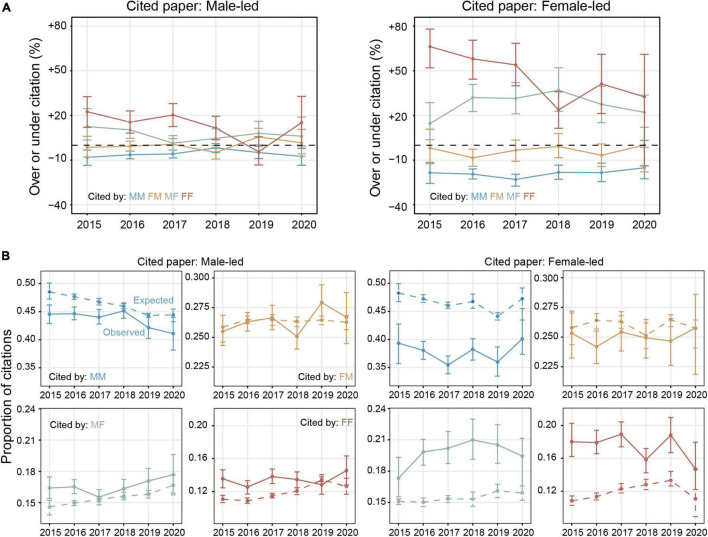
Time-trends in citation rates across gender of the cited and citing author. **(A)** The degree of over-citation and under-citation of male-led and female-led papers over time. The gaps of MM, FM, MF, and FF citations between observed and expected proportions were increasing at a rate of 0.13, 0.60, –1.31, and –1.47% per year within male-led cited papers compared to 0.67, 0.39, 1.45, and –6.74% per year within female-led cited papers. The points represent overall over-citation and under-citation. **(B)** The observed (solid line) and expected (dashed line) proportions of citation within male-led and female-led citing papers over time. The points represent the observed or expected citation rates during a given year. Error bars represent the 95% CI of each rate, which were calculated from 1,000 bootstrap resampling iterations.

After splitting by gender of cited author, we found that the gaps between observed and expected proportions of MM citing papers were increasing at a rate of 0.13% per year within male-led cited papers, compared to 0.67% within female-led cited papers (Figure 3A; observed and expected proportion across citing groups are shown in Figure 3B). In fact, the gaps in MM citation within female-led papers over time have been increasing faster than that within male-led papers. This finding is in contrast with *Hypotheses 3*, i.e., the proportion of MM citations of female-led papers will be increasing faster than that of male-led papers over time.

### The Relationship Between Social Network and Citation Behavior

So far, we have shown that MF and FF citing papers cite female-led cited papers more often than expected (i.e., prediction from our model), whereas MM and FM papers cite less frequently than expected. One question is whether researchers are more likely to work with others of their own gender in the ophthalmology area as the findings by Ghiasi et al. (25) indicate. This could be addressed by examining the social network analytics and co-author relationship networks (26).

**FIGURE 4:**
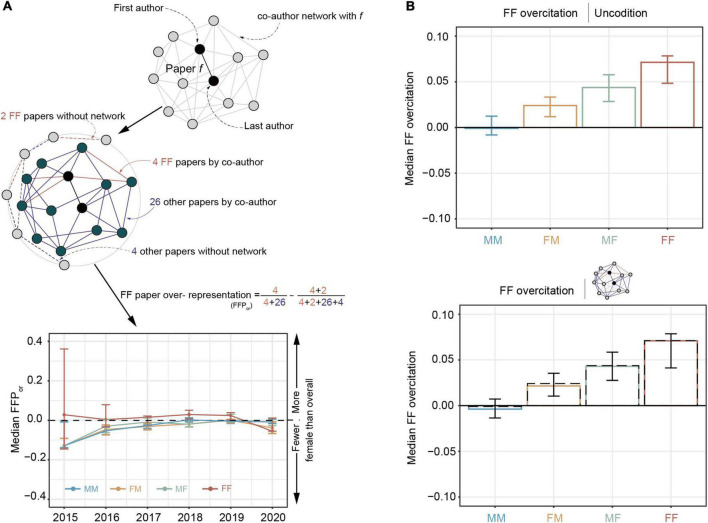
Display of the composition measurement of the co-author network. **(A)** Examples of calculation of the FF paper overrepresentation (FFP_or_) for the specific paper, and the median FF papers in the local network composition based on author gender from 2015 to 2020. **(B)** Over-citation of FF papers without network (the bar graph above and the dashed graph below) and after accounting for network (the bar graph below) influence. The bar graphs represent median FF papers of different gender groups (MM, FM, MF, and FF). The error bars represent the 95% CI of each estimation, which was calculated from 1,000 bootstrap resampling iterations There is only a slight median difference in the citation behavior (MM, –0.001 and –0.004; FM, 0.024 and 0.022; MF, 0.044 and 0.043; and FF, 0.071 and 0.071).

For a given paper *f*, we defined FF paper’s overrepresentation as the difference between the FF papers within *f*’s paper neighborhood and the overall proportion of FF papers within the network at the time of *f*’s publication. Specifically, the number of papers published before the given paper *f* is *n*, where the number of FF papers is FF_all_; the number of papers forming a co-author network with *f* is *m*, the number of FF papers is FF_net_, and the FF paper overrepresentation means $\frac{F\,F_{n\,e\,t}}{m}-\frac{F\,F_{a\,l\,l}}{n}$. As shown in Figure 4A, co-authorship networks tended to include fewer FF papers than the base rate in the field, and overrepresentation of FF papers also differed based on the author’s gender and time. Co-authorship networks tended to cite fewer FF papers than the base rate in the overall field, but this underrepresentation phenomenon has improved over time. In this case, the median FF papers were roughly overrepresented relative to the field’s base rate within the networks of MM teams (− 0.04; 95% CI, − 0.09 to 0.02), FM teams (− 0.04; 95% CI, − 0.09 to 0.00), FM teams (− 0.03; 95% CI, − 0.08 to 0.02), and FF teams (0.01; 95% CI, − 0.03 to 0.04).

Furthermore, we checked whether the composition of the author’s social networks accounts for the citation behavior of women. We utilized the absolute difference of FF citations between the observed proportion and the expected proportion based on the GAM. We found that, without social network, the median MM teams cited fewer FF papers by 0.1% (95% CI, − 0.8 to 1.2%, *p* = 0.84), whereas they cited more FF papers by 2.4% for FM teams (95% CI, 1.2–3.3%, *p* < 0.001), 4.4% for MF teams (95% CI, 2.8–5.8%, *p* < 0.001), and 7.1% for FF teams (95% CI, 4.8–7.8%, *p* < 0.001; Figure 4B).

However, after the social networks have been accounted for, the gender citation patterns remain. Specifically, the median MM teams still cited FF papers less by around 0.38% (95% CI, − 1.4 to 0.8%, *p* = 0.47), whereas they cited FF papers more by 2.2% for FM teams (95% CI, 1.33.7%, *p* = 0.001), 4.3% for MF teams (95% CI, 2.7–5.6%, *p* < 0.001), and 7.1% for FF teams (95% CI, 4.3–7.9%, *p* < 0.001). The citation behavior of women by other women remains after accounting for social networks (*p* = 0.93). These two results do support *Hypothesis 4*: There seems to be a relationship between local co-authorship networks and citation behavior.

## Discussion

We found that the overall proportion of female-led papers increased slightly from 2015 to 2020, but the proportion was only 30% in 2020. Detailed analyses indicate that our finding does not confirm *Hypothesis 1:* The citation rate of female-led papers is lower than expected. In fact, after considering the related characteristics of papers, we found that the proportions of citation of male-led papers were lower than expected and that of female-led papers were higher than expected. However, we found that MM papers cited other MM, FM, MF, and FF papers less by 1.6, 13.5, 16.1, and 23.5%, respectively, compared to their expected proportions. For FF papers, these values were, respectively + 6.8, +29.5, +40.0, and +57.7%. It means that MM papers have under-citation compared to female-led papers. This finding agrees with *Hypothesis 2:* The citation of female-led papers occurs to a fewer degree in MM citing papers. The over-citation rate of male-led papers will be growing slower than that of female-led papers over time, which is contrary to *Hypothesis 3:* The proportion of MM citations of female-led papers will be decreasing more than that of male-led papers over time. Our findings also agree with *Hypothesis 4:* A relationship exists between local co-authorship networks and citation behavior.

Our conclusions regarding *Hypotheses 1 and 3* are different from those in a previous study that investigates citation behavior in the field of neuroscience field (21). It may be because scientists have put more effort into balancing the gender gap in the field of ophthalmology. For example, the proportion of women as first or last authors (10, 27), the number of women holding important positions (4, 13, 14), and the number of women winning awards (28) have increased. A comparison of the findings with those of other studies confirms that men are less likely to cite papers written by women (29–31). Similarly, we found that female scientists in the field of ophthalmology also tended to cite papers written by male authors less frequently. The result of co-authorship networks analysis may provide some support that women may consciously look for and cite work by other women to fight gender imbalance. These findings might explain how our conclusions regarding *Hypotheses 2 and 4* are the same as those in the previous study that investigates citation behavior in the field of neuroscience (21).

As we all know, gender equity is not a short-term job. Since the proportion of FF papers is much lower than that of MM papers, even if the FF papers cited more female-led papers, the gender gap in citation behavior might not be alleviated. It is an important step to improve the willingness of researchers, especially men, to address the existing gender imbalance. Furthermore, addressing the current gender imbalance in ophthalmology can appropriately increase the proportion of women in senior positions and then encourage women to publish more scientific creations (32). It has also been pointed out women are consistent with men in publishing papers early in their careers (33), but women still contend with an excessive burden of family responsibilities (15, 34), resulting in reduced outcomes after they get married. To address these identified imbalances, society should encourage men to bear family responsibilities.

### Limitation and Future Work

Although our study reduces the confound of journal prestige, we still agree that it does not capture the entirety of the field as we selected only four journals. We aimed to evaluate the extent of gender differences in citations to the ophthalmology literature, so the h-index of the journal was the main index for selecting target journals (35, 36). Based on the similar study in this area (21) and limited by resources, we only selected three research journals (*Ophthalmology*: 244; *Invest Ophthalmol Vis Sci*: 218; and *JAMA Ophthalmol*: 196) and one review journal (*Prog Retin Eye Res:* 152) with the highest h-index in the ophthalmology field in 2020. Furthermore, we collected more than 8,000 cited papers from these four journals, and more than 50,000 papers citing them across the whole PubMed database. Even though we believed that these datasets could be a good representation of the question we asked, we agree that an ideal way should be to consider all the journals. Further studies should explore the gender gap in citation behavior by examining more ophthalmology journals.

The previous studies on the gender bias in ophthalmology focus mainly on authors’ characteristics, such as the percentage of female authors (10, 27), female academic ranks (9, 14), and citation count (12). These previous reports provide valuable information on the consequences of gender behavior. In this study, we used the framework of the relationship between authors’ gender and the gender makeup of their cited-by lists to directly measure the citation behavior itself. Limited by the resources we collected from PubMed, we did not include the author’s characteristics (i.e., authors’ publication count, age, and academic rank) into our GAM model. We conducted citation gender analysis through papers’ characteristics rather than authors’ characteristics, not only because it has been validated in the neuroscience area but also because we used the same model for analyzing the gender bias as Dworkin et al. (21). On the other hand, it is also hard to clearly define authors’ characters through the PubMed database. To illustrate, an author might have cross-academic backgrounds; therefore, we do not have an ideal way of determining whether he/she belongs to ophthalmology or other fields. Early studies define the research disciplines of authors based on departmental affiliations (37). However, given the development of interdisciplinary collaboration, particularly to the diversity of researchers’ backgrounds, authors may not be easily classified by a single field (38). Nevertheless, we believe that the citers (people who cited the references) might not really look into the author’s gender before deciding to cite it or not. Thus, the citation bias that we found in the current study could be a reflection of women facing more gender-related obstacles in career development.

There are multiple avenues by which future works could be undertaken based on our study. For example, the paper citation might differ in different ophthalmology subfields, such as retina, cataract, glaucoma, and strabismus. Therefore, it would be interesting to further assess the difference between the ophthalmic sections in the gender gap in citation counts. Limited by the resources we got from the PubMed database, we were not able to extract the keywords and classify the papers into these different categories. Future work may combine other databases into the GAM model to address it.

## Conclusion

In summary, despite the increase in the proportion of female-led papers from August 2015 to July 2020, the proportion was still found to be much lower than that of the male-led papers. Since the proportion of FF papers is much lower than that of MM papers, even if the FF papers cited more female-led papers, the gender gap in citation behavior might not be alleviated; this phenomenon might be related to social co-authorship networks.

## Data Availability Statement

The raw data supporting the conclusions of this article will be made available by the authors, without undue reservation.

## Author Contributions

SC and JZ designed the study. SC, YX, and WZ collected the data. SC, ZH, and JZ contributed to the interpretation of the results and critical revision of the manuscript for important intellectual content and approved the final version of the manuscript. All authors have read and approved the final manuscript.

## Conflict of Interest

The authors declare that the research was conducted in the absence of any commercial or financial relationships that could be construed as a potential conflict of interest.

## Publisher’s Note

All claims expressed in this article are solely those of the authors and do not necessarily represent those of their affiliated organizations, or those of the publisher, the editors and the reviewers. Any product that may be evaluated in this article, or claim that may be made by its manufacturer, is not guaranteed or endorsed by the publisher.

## Footnotes

1
http://genderapi.io/
